# Multi-Scale, Direct and Indirect Effects of the Urban Stream Syndrome on Amphibian Communities in Streams

**DOI:** 10.1371/journal.pone.0070262

**Published:** 2013-07-29

**Authors:** Stefano Canessa, Kirsten M. Parris

**Affiliations:** ARC Centre of Excellence for Environmental Decisions and the NERP Environmental Decisions Hub, School of Botany, University of Melbourne VIC 3010, Australia; University of California, Berkeley, United States of America

## Abstract

Urbanization affects streams by modifying hydrology, increasing pollution and disrupting in-stream and riparian conditions, leading to negative responses by biotic communities. Given the global trend of increasing urbanization, improved understanding of its direct and indirect effects at multiple scales is needed to assist management. The theory of stream ecology suggests that the riverscape and the surrounding landscape are inextricably linked, and watershed-scale processes will also affect in-stream conditions and communities. This is particularly true for species with semi-aquatic life cycles, such as amphibians, which transfer energy between streams and surrounding terrestrial areas. We related measures of urbanization at different scales to frog communities in streams along an urbanization gradient in Melbourne, Australia. We used boosted regression trees to determine the importance of predictors and the shape of species responses. We then used structural equation models to investigate possible indirect effects of watershed imperviousness on in-stream parameters. The proportion of riparian vegetation and road density surrounding the site at the reach scale (500-m radius) had positive and negative effects, respectively, on species richness and on the occurrence of the two most common species in the area ( *Crinia* *signifera* and *Limnodynastesdumerilii* ). Road density and local aquatic vegetation interacted in influencing species richness, suggesting that isolation of a site can prevent colonization, in spite of apparently good local habitat. Attenuated imperviousness at the catchment scale had a negative effect on local aquatic vegetation, indicating possible indirect effects on frog species not revealed by single-level models. Processes at the landscape scale, particularly related to individual ranging distances, can affect frog species directly and indirectly. Catchment imperviousness might not affect adult frogs directly, but by modifying hydrology it can disrupt local vegetation and prove indirectly detrimental. Integrating multiple-scale management actions may help to meet conservation targets for streams in the face of urbanization.

## Introduction

In 2010, more than half the world’s population was living in cities, a proportion predicted to reach 70% by 2050 [1]. The processes of urbanization, described as the change in land use from natural or agricultural to residential or industrial, comprise many of the major recognized threats to biodiversity [2]. Human activities in urban areas produce direct mortality and pollution, both chemical and physical [3,4]. The field of urban ecology has been developing over the last 30 years in an effort to provide quantitative descriptions of these impacts [2].

Given that most cities have developed in close proximity to waterways, it is not surprising that the study of stream ecology in urban areas has been a particularly proficient field, leading to the definition of the “urban stream syndrome” [5]. Streams in urban areas show an increased flashiness of storm flows: rainwater runs off the high proportion of impermeable surfaces in the watershed (roads and buildings) and is fed directly into the stream along storm-water drainage systems, rather than percolating through the soil, recharging the water table [5]. This can accelerate erosion and input of polluting substances [6]. Direct modification of the water course, such as forced channelization, is also common [7].

The effects of these changes on fish and invertebrate species with aquatic life stages have been thoroughly investigated [8,9], while impacts on other taxa such as plants and diatoms have also been studied [10]. In general, increasing urbanization has negative effects on sensitive and specialist taxa, whereas tolerant generalists may increase until the degree of modification becomes excessive [11]. However, the exact shape of the response is still unclear. Early studies tended to estimate negative responses above a threshold of 10% of land in the watershed converted to urban use [12], but more recent analyses have suggested lower or no thresholds, with negative effects even at very low levels of urbanization [13,14]. There has also been a progressive shift from single-system, single-scale analyses to more complex investigation of multiple-scale effects [9,15].

These changes reflect developments in the general theory of stream ecology, such as the Riverine Ecosystem Synthesis (RES) [16]. It is now generally accepted that the stream cannot be analysed separately from its watershed, and that the relationship between dynamics along the entire stream and at the local scale is influenced by processes within the stream, across the landscape and in time: landscape use at the watershed scale ultimately affects the in-stream ecosystem [17]. This is also true for urban streams, which may be affected by modifications of the surrounding landscape, regardless of the reach- and local-scale quality of the main channel and riparian zone [14]. At the same time, local modifications (channelization, vegetation removal) may determine a patchy discontinuity in habitat quality that goes beyond the variation that could be expected when interpreting the stream as a continuous longitudinal gradient.

As interactions between the riverscape (main channel and slackwaters) and riverine landscape (inundation zones: definitions from the RES) receive more attention, studies of animal ecology may increasingly focus on taxa that provide a functional link between the two levels. A number of previous studies have investigated the response of bird, mammal and amphibian communities to urbanization [18,19]. Amphibians in lentic water bodies are negatively affected by changes to pond structure, vegetation and hydroperiod [20–22], by traffic noise [4], and by the introduction of invasive predatory fish [23]. Recent studies have found urbanization-related modifications of streambed and hydrology negatively affect larval salamanders, providing a first explicit link to the urban stream syndrome [24,25]. However, stream amphibians in general, and anurans in particular, have been under-represented in the urban ecology literature (but see 26,27).

Amphibians with semiaquatic life cycles serve as an important link in the energy flow between the stream and the surrounding landscape, a concept relevant to stream ecology since the introduction of the river continuum concept [28]. Adult amphibians occur and move across the riverscape and riverine landscape, and are likely to be affected, directly or indirectly, by urbanization processes at different scales, from local breeding sites to reach-scale riparian areas and upland watershed sections. Understanding these effects, already demonstrated for non-urban areas [29], may provide further details regarding the urban stream syndrome within the general framework of stream ecology.

In this study, we focused on anuran communities along a gradient of urbanization in Melbourne, Australia. We related the species richness of anuran assemblages and the probability of occurrence of individual species to measures of urbanization at different spatial scales. We sought to (1) identify the elements of urbanization that most affect frog assemblages and individual species; (2) assess the shape and magnitude of these relationships and compare them with existing knowledge of both urban stream communities and anurans in urban areas; and (3) identify interactions and indirect effects between variables across multiple scales, and interpret these in light of the current knowledge of urban streams and broader stream ecology. We found relevant negative effects of urbanization on frog communities, with direct effects particularly strong at the local and reach scale (<500 m) and possible indirect effects of watershed-scale modification, affecting frogs by modifying local site features.

## Materials and Methods

### Study area and site selection

**Figure 1:**
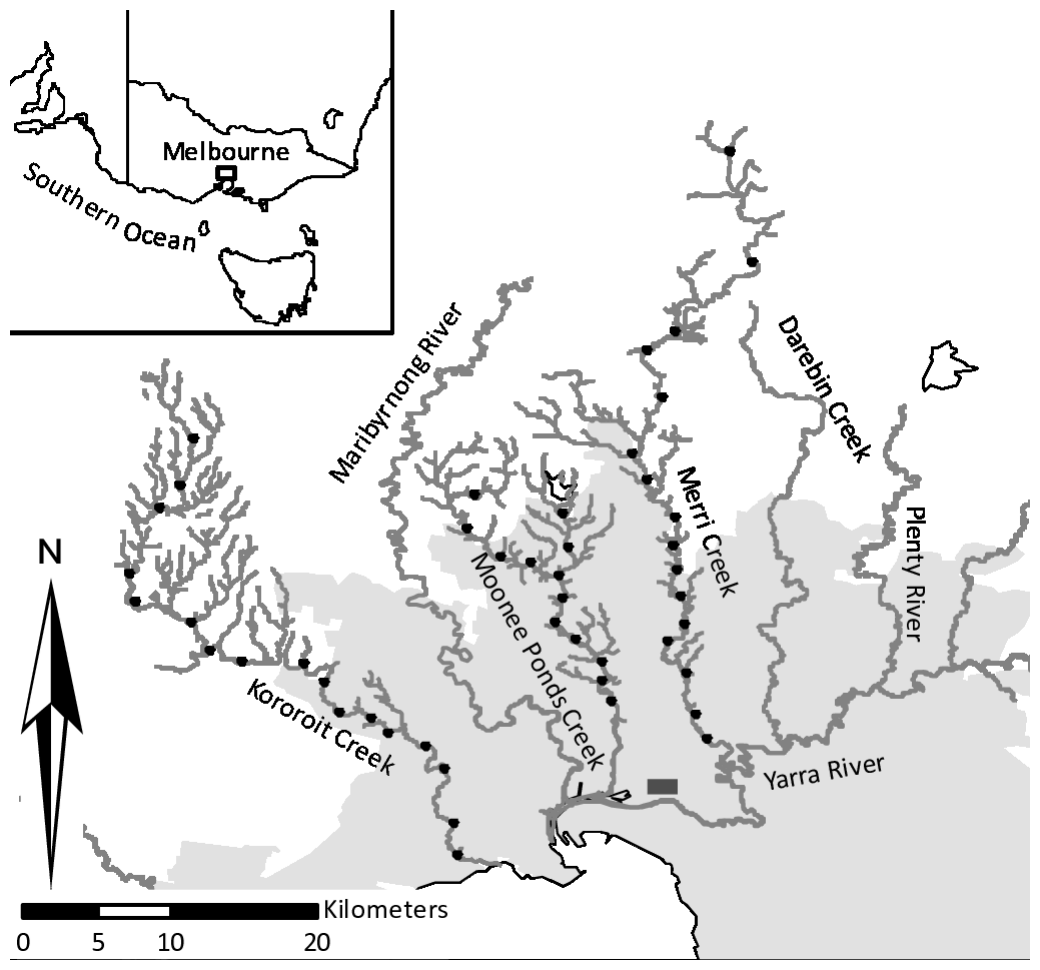
Map of the study area. Circles indicate the location of survey transects. The grey area represents the extent of current development, with the dark grey rectangle indicating the central business district of the city. Waterways in black.

Our study focused on three small streams, the Kororoit, Moonee Ponds and Merri Creeks, that run approximately north–south through the western and north-western suburbs of Melbourne, with a catchment area of approximately 1,000 km^2^ (Figure 1). All three creeks follow a general gradient of urbanization, each one flowing from rural and natural areas close to their headwaters, through increasingly developed areas in the lower reaches, although with some local discontinuity. To quantify the landscape-scale gradient of urbanization along each creek and ensure it was captured in our site selection, we selected a simple measure of attenuated imperviousness (AI). This corresponds to the total area of impervious surfaces in a subcatchment (the segment of stream between two confluence points), weighted with exponential decay and a half-decay distance of 9.2 m from the nearest drain (for an explanation of this value see 30).

We chose AI as the core watershed-level predictor of the impacts of urbanization on stream frog communities for a number of reasons. It reflects the main hydrological modifications to urban streams, particularly flashiness of floods and increased base flow, accounting for connectedness of impervious areas to drainage systems, and it has been shown to correlate negatively with the diversity and composition of fish and invertebrate communities in a number of studies, including several in the Melbourne region [8,10,14,30,31].

Within our study area, AI increases in similar fashion along each stream, with higher values in the lower reaches of the Moonee Ponds creek. Within our three focal watersheds, AI varies between 0 and 18% and is strongly correlated with distance downstream (Pearson’s *r*=0.89±0.2). To capture gradient variation, we classified AI in fifteen classes for each creek and selected one 200-m transect per class, ensuring no pair of sites shared the same subcatchment. To minimize chances of spatial autocorrelation between sites (unexplained spatial structure in species data) and movement of frogs between sites during the sampling period, we also set a minimum between-site distance of 1 km (Euclidean) and of 1.5 km along the stream network, to account for the fact that stream corridors may facilitate frog movements. The average dispersal distance for the smallest frog in the area, the common froglet *Crinia* *signifera* , has been estimated to be around 500 m in semi-natural habitats [32]. After correcting for minimum distances and obtaining access to sites, we defined a final set of 46 transects (Figure 1).

### Field methods

To optimize the accuracy of species surveys, we estimated *a priori* the probability of detecting adult male frogs using standardized aural surveys (methods and data described in [33,34]). For the species we expected to occur in the study area ( *Crinia* *signifera* , *Limnodynastesdumerilii* , *Limnodynastes* *peroni* , *Limnodynastes* *tasmaniensis* , *Litoriaewingi* , *Litoria* *peroni* , *Litoriaraniformis* , *Litoria* *verreauxi* ), we estimated that three 30-minute nocturnal visits per site, between late September and early December, would ensure a probability of detecting most species, if present, of more than 90% (no reliable estimates could be produced or sourced for *Litoria* *peroni* and *Limnodynastes* *peroni* ). Accordingly, all sites were visited three times, for a total of 138 surveys, between late September and late November 2009. We walked the transect slowly, recording any call heard while walking and in 5-minute static listening periods every 50 m. Frog species in the area have distinctive mating calls, minimizing the chance of misidentification [35]. To avoid observer effects, all surveys were carried out by the main author, assisted by one of a panel of field assistants. Data collection was approved by the Animal Ethics Committee of the University of Melbourne (project ID 0911299.1). Approval from the Department of Sustainability and the Environment (Victoria) was sought and obtained without the need for a permit, on the premise that the study was observational only and no handling of animals was required. Field sites located on private land were accessed after obtaining permission by land holders. No sites were located within protected areas.

To assess the relative influence of local-scale impacts and watershed-scale modifications (reflected by imperviousness), we also selected and measured a range of environmental variables at smaller scales. At each site, we measured the electrical conductivity of the water using hand-held probes (Oakton meters, Singapore), as a proximate measure of pollution, known to correlate negatively with frog species richness in our study area [20] and with biotic communities in streams worldwide [11]. We recorded the proportion of floating, submerged and emergent vegetation within each transect, and aggregated this into a mean value of “aquatic vegetation”: this measure has been demonstrated to correlate positively with occurrence of several frog species in urban areas in Melbourne [20]. Since the low rates of flow in most transects did not allow reliable measurements of flow velocity, we assessed the proportion of pool-bed forms (deep, slow-flowing water) within each transect to quantify the proportion of suitable flow conditions for tadpole development. We calculated the proportion of the riparian zone along the stream (a 10 to 30-m wide strip from the stream bank) covered by green open vegetation space within a 500-m buffer around each transect. This variable has also been suggested to influence the presence of frogs in urban ponds in Melbourne [20,21]. We corrected the observed value with GIS data and satellite imagery [36]. At the landscape scale, we also calculated the road density (km/km^2^) within a 500-m buffer around each site, using ArcGIS®. High road densities are known to affect amphibian species negatively, both through direct mortality and chemical, noise and light pollution [37].

### Statistical analyses

Although the number of species at a site can be a poor description of amphibian assemblages [38], it would allow use of a Poisson-distributed response variable, without resorting to complex multivariate indices. Therefore, we initially assessed the correlation between the number of species and community dissimilarity (Bray-Curtis index) using Mantel tests (correlation coefficients and 95% confidence intervals calculated over 100,000 permutations).

We used boosted regression trees (henceforward BRTs) to assess the contribution of environmental predictors in explaining data on the species richness of assemblages and the occupancy of sites by individual species. BRTs fit a large number of simple regression trees (which relate each predictor to the response variable by a series of recursive splits) and combine them using machine learning techniques (boosting), improving predictive performance and allowing representation of complex, nonlinear functions [39]. We used Poisson and binomial error distributions for species richness and species occupancy respectively. The candidate predictor set included electrical conductivity, proportion of pool sections, aquatic vegetation cover, riparian vegetation cover, road density and attenuated subcatchment imperviousness. We calculated pairwise Pearson’s correlation coefficients between variables to assess multicollinearity within the predictor set. Only road density and riparian vegetation had *r*>0.5 (originally *r*=0.54, reduced to *r*=0.48 after log-transformation of road density): all other pairs of variables had *r*<0.4. We fitted all models using package GBM in R [40].

Fitting BRTs requires definition of two parameters: the learning rate (lr) represents the contribution of each tree to the model set, while tree complexity (tc) determines the fitted interactions (tc=1 indicates a simple additive model (decision stump), while tc=2 indicates two-way interactions, and so on). We used sensitivity analysis to determine the optimal balance of learning rate (lr) and tree complexity (tc), by running multiple iterations of the *gbm.step* function [39] under different combinations of values (tc=1, 2 and 3; lr=0.001, 0.0025 and 0.005).

Given the stochasticity in the boosting process, results differ slightly for each model run. Therefore, we ran the *bgm.step* function 1,000 times and calculated the mean number of trees fitted. We assessed predictive performance using ten-fold cross-validation, by comparing the correlation between predicted and observed values for species richness and area under the ROC curve scores (AUC) for species occupancy. We selected for further analysis the learning rate that provided the best predictive performance, while keeping the number of trees fitted between 1,000 and 3,000, to minimize the risk of overfitting [39].

We estimated the contribution of each variable based on how often the predictor was selected, and the improvement it contributed to the model. We then scaled relative influences to sum to 100%: we considered variables with influence greater than 5% to be relevant, while removing those contributing less than 5% in order to simplify the model and avoid overfitting. We investigated the shape of each relationship using partial dependence plots of the effect of each predictor while controlling for the average effect of all other variables in the model. We plotted the mean estimated response (with 95% confidence intervals) over the 1,000 fitting runs. We assessed the relevance and magnitude of interactions between predictor variables for trees with *tc*>1.

### Indirect effects

Transformations at the watershed level can affect in-stream characteristics and influence communities to a greater extent than can be revealed by local-scale models. We therefore used path analysis [41] to evaluate whether the level of imperviousness of the subcatchment affected the local characteristics of the riverscape and could therefore have an indirect effect on species richness. We used JAGS [42] to re-fit a model which included the most influential parameters identified by the BRT analysis (the number of parameters that could be fitted was limited by the sample size). Bayesian models are naturally suitable for multilevel model analysis, and working in a Bayesian framework also allowed us to observe the full probability distribution of the parameters of interest (regression parameters reflect the strength of the indirect and direct effects of AI on local-scale parameters and of both variables on species richness). We fitted one null model with direct effects only, a “mediation” model with an indirect effect of attenuated imperviousness on reach- and local-scale characteristics only, and a saturated model including direct and indirect effects of AI. Within each model, we accounted for unmodelled variability among streams by including a categorical random effect for the factor “creek”.

Using uninformative priors, for each model we ran 100,000 iterations on three separate Markov chains with overdispersed starting values, discarding the first 50,000 iterations as a burn-in. We standardized all variables to allow direct interpretation of relative effect sizes (correlation coefficients). Models were compared using the deviance information criterion (DIC [43]:). DIC values reflect a trade-off between the fit of a model and its complexity, with smaller values indicating a better-supported model. When comparing models, those with DIC values within 2 points of each other can be considered to be largely indistinguishable. We investigated the JAGS output for the distribution of each parameter to interpret the statistical effect of predictors on the response variable, with 95% credible intervals. We then used the regression coefficients to construct and interpret path diagrams for each model configuration: we calculated indirect and direct effects as the products of all regression coefficients along a given pathway [41].

### Spatial autocorrelation

In the site-selection stage, we set a minimum distance between sites of 1 km (Euclidean distance) and 1.5 km (along the stream network), based on the known average movement distances of frog species in the area. However, it can be assumed that individual frogs will occasionally move much further than the average distance [44]. Therefore, we could not rely on study design alone to ensure the absence of spatial autocorrelation in our collected species data. If movement did occur between sites, particularly those along the same stream, then one would expect species richness or occupancy to be determined by spatial factors beyond the environmental predictors considered.

To investigate this possibility, we assessed the residual spatial structure in compositional data and environmental variables that was not explained by the model. After refitting the models in JAGS, we calculated the semivariance of paired residuals for the “null” model (direct effects only) and its coefficient of correlation with pairwise distances between sites, measured as both Euclidean distance and stream-network distance. A large correlation coefficient would indicate that unexplained variation in the model may be the result of unmodelled spatial processes, such as long-range dispersal [45]. Conversely, a small correlation coefficient would suggest the spatial structure in the data is entirely explained by the spatial patterns in the model predictors.

## Results

We detected seven species of frogs during our surveys. Thirty-four of 46 sites were occupied by at least one species, with a maximum of six at one site. *Crinia* *signifera* was the most common species, detected at 27 sites, while *Limnodynastesdumerilii* was detected at 18 sites. The other species were less common ( *Limnodynastes* *tasmaniensis* : 13 sites, *Litoriaraniformis* : 9, *Litoria* *ewingii* : 8, *Limnodynastes* *peronii* : 1, *Litoria* *verreauxii* : 1). Therefore, we fitted single-species models only for the two most common species. Given the simple community composition, with most sites sharing the two or three most common species, we considered species richness to be a good measure of assemblage similarity (Mantel correlation coefficient *r*=0.72, 95% CI: 0.68, 0.76), and therefore retained it as a response variable. Sensitivity analysis of BRTs showed that a learning rate of 0.0025 allowed us to fit at least 1,000 trees for all response variables. A tree complexity of 1 gave insufficient predictive performance, while increasing tree complexity from tc=2 to tc=3 did not improve performance. Given the small sample size, we considered a final configuration of lr=0.0025 and tc=2 to be the most parsimonious for all models fitted.

**Figure 2:**
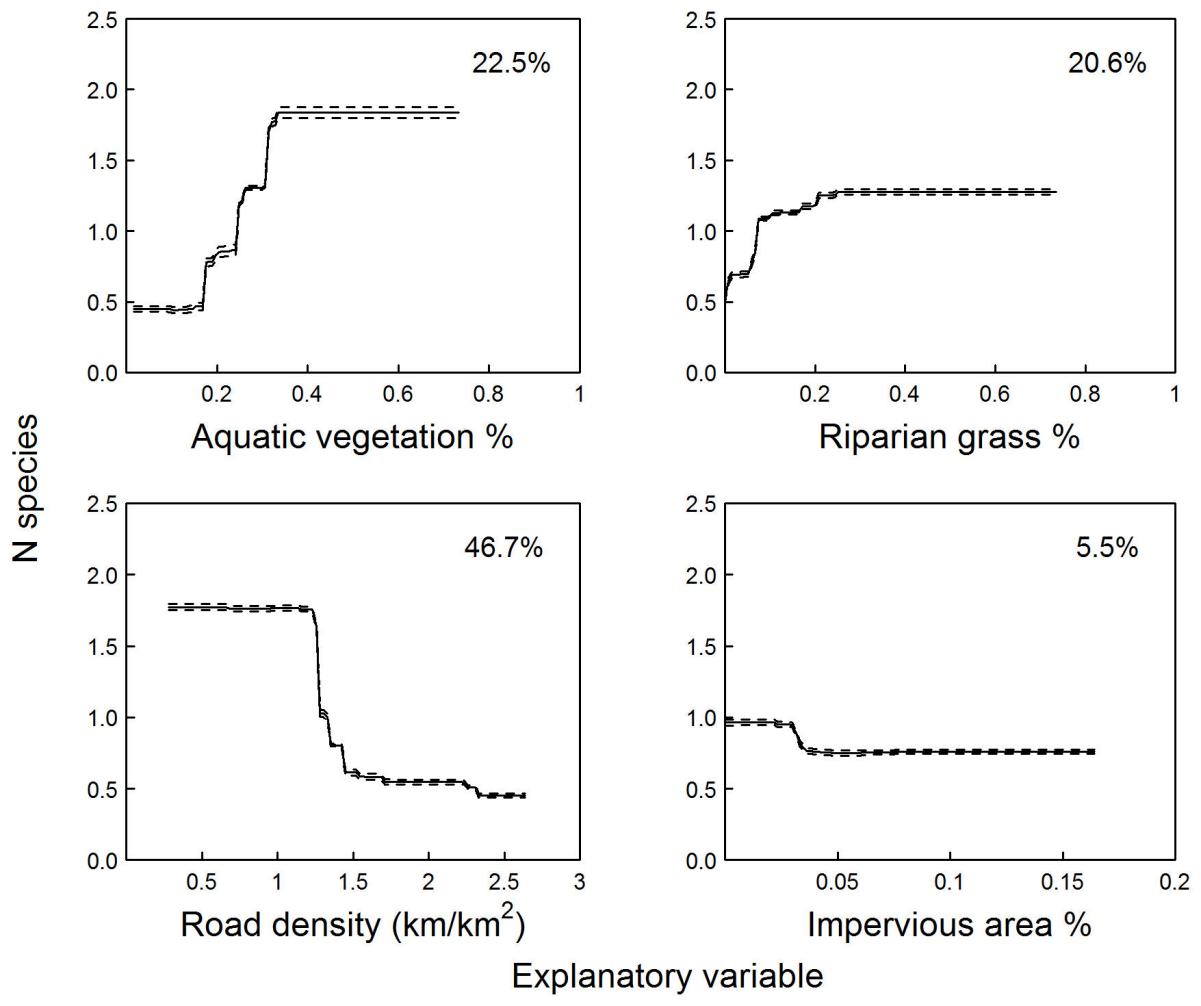
Marginal effect plots of the three main explanatory variables (deviance explained >5%). Plots represent the effect of each variable on species richness (top) and probability of occupancy for *C* *. signifera* (centre) and *L* *. dumerilii* (bottom) while controlling for the average effect of all other variables in the model. Road density values log-transformed. Dashed lines represent 95% confidence intervals calculated over 1,000 iterations of the model fitting function.

**Figure 3:**
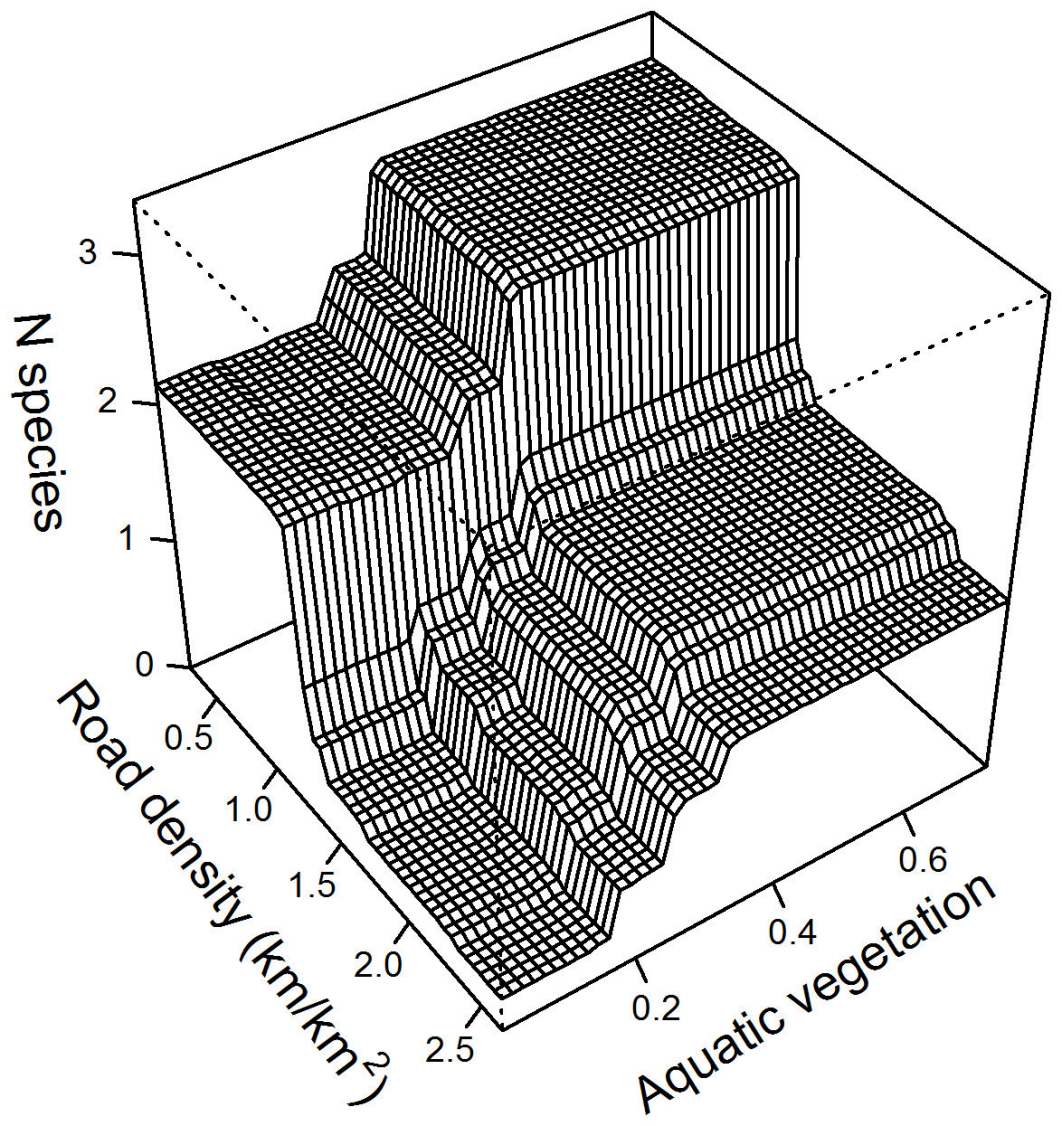
Interaction between road density and aquatic vegetation in influencing species richness. Road density values log-transformed on the x-axis.

For species richness, the average cross-validation score over 1,000 runs was 0.71 (±0.002 s.d.), with a mean number of 1,275 trees fitted. Road density provided the greatest contribution to the model (deviance explained D=46.7%). A sharp decline in the predicted species richness was observed between 2.7 and 4.5 km/km^2^ of road density within 500 m from the site, with little response outside this range (Figure 2; note plot values are log-transformed). Aquatic vegetation (D=22.5%) had a positive effect on species richness, with an increase between 20% and 40% site cover, while proportions of cover greater than 50% did not show significant additional responses. Riparian vegetation (D=20.6%) had a positive but limited effect on species richness, with an appreciable decrease at sites with less than 20% cover, and little response above this value. The proportion of impervious area within the catchment accounted only for 5.5% of the total deviance explained, and its effect appeared limited to a small negative response at low levels (less than 0.05 cover). Electrical conductivity and the proportion of pool sections within each transect did not contribute significantly to the model (D<5%), and their removal did not affect predictive performance. We therefore removed them from the final version fitted. We found a significant negative interaction between road density and aquatic vegetation: the positive effect of denser vegetation was reduced at sites with high surrounding road density (Figure 3).

For *Crinia* *signifera* , an average 1,854 trees were fitted (tc=2, lr=0.0025), with a final mean AUC score of 0.86 (±0.001). Riparian vegetation was the most influential predictor (D=74.6%), showing a strong positive effect on the probability of occupancy by the species, which increased from 0.2 to 0.8 for cover proportions between 0 and 0.35, while further increases did not show any effect (Figure 2). The proportion of impervious area in the subcatchment and road density around the site contributed less to the model (D=11.5% and D=6.9% respectively), showing non-linear, limited marginal effects. Aquatic vegetation had a positive effect on the probability of occupancy, but the magnitude of the effect was small and this predictor contributed to explain just 7% of the total deviance. Electrical conductivity and the proportion of pool sections within the channel explained less than 1% of the total deviance and we therefore removed them from the final model. We detected no important interaction between predictor variables.

Models for *L* *. dumerilii* were fitted using an average of 2,250 trees. About 150 of the 1,000 runs for this species did not converge adequately, so results should be interpreted with some caution. However, when models converged the average predictive performance was good (AUC=0.93±0.001). Aquatic vegetation was the most influential predictor (D=62.8%) and showed a strong threshold effect: the probability of occupancy by the species increased from 0.1 to 0.87 when increasing the proportion of aquatic vegetation cover from 0.25 to 0.3 (Figure 2). Subcatchment imperviousness (D= 20.9%) had a negative influence on occupancy, which decreased from 0.6 to 0.1 as soon as imperviousness departed from zero (AI<0.05). Electrical conductivity and road density explained some of the model deviation (D=10.3% and D=6.0% respectively), with a nonlinear response of difficult interpretation. The proportion of pool sections and riparian vegetation explained less than 1% of the total deviance and were therefore removed from the final model fitted. We found no important interaction between predictors.

### Indirect effects

**Figure 4:**
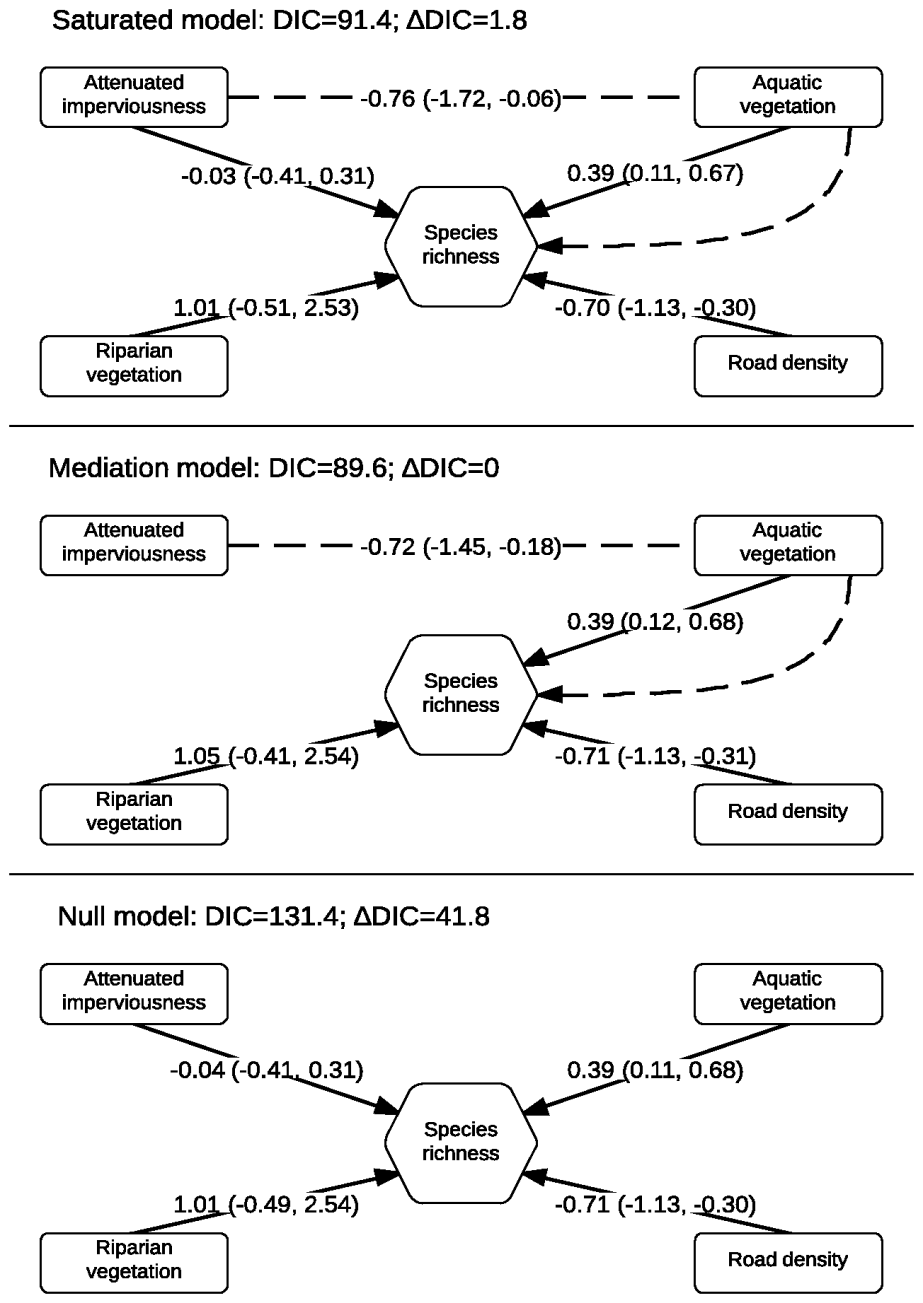
Path diagrams for the structural equation models fitted to species richness data. The saturated model (top) includes both direct effects of attenuated imperviousness (AI) and indirect effects through modification of aquatic vegetation. The mediation model only incorporates indirect effects of AI (centre), whereas the null model only includes direct effects for all predictors (bottom). DIC is the deviance information criterion and ∆DIC the difference between DIC scores of the best model and a given model. The dashed arrow represents the total indirect effect of attenuated imperviousness on species richness (by affecting aquatic vegetation). Solid arrows indicate direct effects. Numbers next to arrows indicate the standardized coefficient for that path (95% credible intervals in brackets).

Given the limited sample size, we restricted the second step of the analysis to species richness and occurrence of *C* *. signifera* . We refitted the model in JAGS, including relevant predictors highlighted by BRT analysis: aquatic and riparian vegetation, road density and impervious area. We assessed the hypothesis that AI would impact species richness indirectly by negatively affecting aquatic vegetation through altered flow regime (Figure 4).

The mediation model was most supported (DIC=82.5): the total path coefficient indicated a large, negative indirect effect of AI on species richness (*p*_AI_→aqveg→richness=-0.72, 95% CI: -1.45, -0.18; Figure 4). However, the saturated model also received relative support (ΔDIC=2.2), suggesting a direct effect of AI on species richness cannot be excluded, possibly through other unmodelled factors, although the path coefficient for the direct effect was considerably smaller and surrounded by greater uncertainty than the indirect one (β_AI_→richness=-0.04, 95% CI: -0.41, 0.31; *p*_AI_→aqveg→richness=-0.76, 95% CI: -1.72, -0.06). The null model was not supported (ΔDIC=41.8).

For *Crinia* *signifera* , we refitted models in JAGS including effects of aquatic and riparian vegetation and impervious area. We assessed mediation and saturated models including indirect effects of impervious area on riparian vegetation: the limited sample size prevented the inclusion of an additional indirect effect on aquatic vegetation, which also had a small effect on probability of occupancy. The null model including only direct effects of predictors was largely more supported than the alternative models (null: DIC=41.5; mediation: DIC=174.6; saturated: DIC=175.3). The coefficients suggested a large positive effect of riparian grass (β_ripgrass → occupancy_ =3.19, 95% CI: 1.37, 5.80), a small positive effect of aquatic vegetation (β_aqveg_→occupancy=0.90, 95% CI: -0.24, 2.29) and a large negative effect of AI (β_AI_→occupancy=-1.17, 95% CI: 3.14, 0.61), matching the results of the BRT analysis.

Finally, we analysed the residuals of the refitted “null” models to assess the existence of unexplained spatial autocorrelation in the species data. We found no important correlation between the semivariance of the residuals and pairwise distances between sites, either Euclidean or along-stream (Pearson’s *r* < 0.1). This result was repeated when we carried out a separate analysis for each stream. These results suggest the spatial stratification implemented in the study design accounted satisfactorily for spatially-correlated variation.

## Discussion

Our results show a general negative influence of urbanization on species richness and on the probability of occurrence of frog species along the urban–rural gradient in Melbourne, matching observations for lentic water bodies in the same area [20,21] and worldwide [22,25,27]. The number of species recorded was highest in the peri-urban area and decreased in the more densely urbanized reaches of each creek; only two of the fifteen sites in lower reaches (dense urbanization) hosted one species each, with the rest unoccupied. We found an important influence of explanatory variables at multiple scales, supporting the view that both local and landscape-scale processes should be considered when analysing communities along the stream channel.

Species richness was best explained by aquatic vegetation at the local scale, and road density and riparian vegetation at the reach scale. The decrease in vegetation cover below a certain threshold (0.2-0.3) appeared to determine a reduction in the number of species at a site. Most frogs in the area benefit from aquatic vegetation for egg-laying, and their tadpoles rely on vegetation for shelter from predators. In particular, males of *Limnodynastesdumerilii* call to attract females from concealed positions among reeds [35], and this may explain the strong positive influence of aquatic vegetation on the probability of occurrence of this species. Aquatic vegetation is also known to positively influence occupancy by the endangered growling grass frog *Litoriaraniformis* [46] and by *Limnodynastes* *peronii* and *Litoria* *ewingii* in stormwater retention ponds in Melbourne [47].

Reach-scale variables (500 m) were important in predicting both species richness and the occurrence of *Crinia* *signifera* . The proportion of open riparian vegetation was the most influential predictor for *C* *. signifera* : this species is normally abundant, but its largely terrestrial habits may imply the need for foraging and movement areas (note that the length of the riparian buffer analysed here matches average movement distances for this species observed by Lauck [32]). Riparian vegetation also had positive effects on species richness, matching observations by Hamer & Parris [20] for urban ponds around Melbourne. It can be assumed that most species in the area will rely on terrestrial habitat for foraging and dispersal: these may be affected by fragmentation of continuous riparian corridors following human activities (such as construction). Although some arboreal vegetation may be planted with specific purposes (such as wind screening), the understory is often deprived of vegetation or replaced by artificial surfaces. The importance of maintaining adequate vegetation buffers in the riparian zone on biotic communities in urban streams is well documented [48].

Road density had a strong negative effect on species richness, although its effect on the occurrence of *C* *. signifera* was negligible and the shape of the response of *L* *. dumerilii* was difficult to interpret. Traffic can affect amphibians directly, by causing adult mortality [49]. More recent studies have also pointed out the indirect effects of roads, in particular the traffic-generated noise that may affect the ability of males to attract females for reproduction [4]. We observed a strong negative effect at medium-low levels of road density: generally, increasing road density will be correlated with higher traffic volumes and greater road-traffic noise. We found that road density and aquatic vegetation interacted in influencing species richness: sites surrounded by a high density of roads may be difficult to colonize even though the in-stream habitat appears to be suitable. Similarly, Eigenbrod et al. [50] found that the negative effect of road density offset the positive effect of forest cover on pond amphibians in Canada. Roads can isolate populations and decrease the chances of successful colonization of unoccupied sites [21].

At the landscape scale, the proportion of connected impervious areas in the sub-catchment had only marginal direct effects on species richness and on occupancy of *C* *. signifera* , although it was a relevant predictor for the probability of occurrence of *L* *. dumerilii* (for which negative effects were observed for proportions of AI above 0.01). Although the direct effect of catchment conditions on adult frog communities may contribute little to the model and appear marginal at first, both stream-ecology theory and current knowledge of urban streams suggest that transformations at the catchment scale eventually affect the riverscape and the biodiversity it supports [14,16,17]. Within the urban stream syndrome, altered flow regimes are the direct consequence of an increase in catchment imperviousness [5]. For example, a recent study [51] showed that in the greater Melbourne area, streams connected to conventional urban stormwater drainage systems (such as the streams in our study) suffered severe alterations in flow patterns that were directly related to an increased frequency and flashiness of floods. High levels of connected imperviousness increased the magnitude of flow events by 2-3 times even after small rainfall events.

The resulting flashiness of floods can directly affect aquatic organisms, as widely demonstrated by its negative effects on invertebrates with at least partially aquatic life stages [8,14]. Such events may also cause direct or indirect disturbance to frog species, by disrupting breeding activities for males that call from the water, washing away eggs, or removing vegetation that may have provided shelter from predators for breeding adults, eggs and tadpoles. Recently, Barrett et al. [25] demonstrated substantial effects of altered flows on larvae of stream salamanders, and suggested that these may combine with modification of the substrate to prevent species from surviving at sites. Anuran tadpoles may also be affected, particularly species that are not adapted to fast-flowing water, as is the case for species in our study area [52]. Drainage-related peak flows that occur after minor rain events are more frequent during the tadpole development period (September to March for species in the Melbourne area), at a time when these impacts would be smaller in streams with more natural flow patterns.

On the other hand, adult frogs of the species described in our study are less likely to be directly affected by high peak flows. However, we found an important effect of subcatchment imperviousness on the proportion of aquatic vegetation at a site: sites within more permeable subcatchments were more likely to retain higher vegetation densities, which in turn may favour frog species. This may also explain the relevance of catchment imperviousness as a predictor for *L* *. dumerilii* , which appears to rely on aquatic vegetation, although the small number of observations prevented estimation of an indirect effect for this species. Our results did not suggest an important indirect effect of AI on *C* *. signifera* through disturbance of riparian vegetation (the main predictor of occurrence). It is possible that, in the small streams analysed, peak flows, while impacting in-stream conditions, are not sufficient to significantly alter the riparian inundation zone, in particular given that our study followed several years of drought and near-drought conditions in the area.

Catchment imperviousness may also affect other in-stream parameters, such as water quality and bed-form structures, as described in the existing literature [53]. For example, Riley et al. [27] found that the sequence and proportion of pool and riffle sections influenced amphibian communities in urbanized watersheds in California streams. However, these predictors did not appear to have a relevant influence in our study. On the other hand, tadpoles of the species that occur in our study area, being strictly aquatic, might be indirectly affected. These should be the focus of future research, particularly since tadpoles can often fulfil the role of grazers in streams [54,55], and this is generally true for tadpoles in our study area [52]. Although their diet can also incorporate a significant heterotrophic component [56], grazing tadpoles are particularly important for the river energy cycle in those areas with high production of periphyton (such as urban streams, where algal blooms are common: see 57), as first recognized by the river continuum concept [28]. Anuran larvae generally differ from the salamander taxa which have been the main focus of previous studies of amphibians in urban streams [25,58], as salamander larvae are predominantly carnivorous. Therefore, the impacts of urbanization on species whose tadpoles act as grazers in stream assemblages could determine different ecological effects, and would warrant greater attention in future studies.

Metropolitan Melbourne, as many cities worldwide, is set to expand further over the coming decades [59]. When planning development of new urban areas, offset actions are often undertaken, such as construction of new wetlands to replace those that are destroyed [60]. Government, volunteer and neighbourhood groups may also implement management actions such as revegetation of riparian zones. However, modifications at the watershed scale may have indirect effects on riverscape conditions, potentially undermining local restoration benefits [14]. Management actions need to account for the connections between riverscape and riverine landscape described by stream-ecology theory and reinforced by empirical studies such as the one presented here, as integration between actions at different scales may help to meet conservation objectives.

## References

[1] United Nations (2007) World Population Prospects: The 2006 Revision and World Urbanization Prospects: The 2007 Revision.

[2] Pickett STA, Cadenasso ML, Grove JM, Groffman PM, Band LE et al. (2008) Beyond urban legends: an emerging framework of urban ecology, as illustrated by the Baltimore ecosystem study. BioScience 58: 139-150.

[3] Longcore T, Rich C (2004) Ecological light pollution. Front Ecol Environ 2: 191-198. doi:10.1890/1540-9295(2004)002[0191:ELP]2.0.CO;2.

[4] Parris KM, Velik-Lord M, North JMA (2009) Frogs call at a higher pitch in traffic noise. Ecol Soc 14: 25-45.

[5] Walsh CJ, Roy AH, Feminella JW, Cottingham PD, Groffman PM et al. (2005) The urban stream syndrome: current knowledge and the search for a cure. J North American Benthological Society 24: 706-723. doi:10.1899/04-028.1

[6] Booth DB, Henshaw PC (2001) Rates of channel erosion in small urban streams. Water Science and Appl 2: 17-38. doi:10.1029/WS002p0017.

[7] Meyer JL, Paul MJ, Taulbee WK (2005) Stream ecosystem function in urbanizing landscapes. J North American Benthological Society 24: 602-612. doi:10.1899/04-021.1

[8] Walsh CJ, Sharpe AK, Breen PF, Sonneman JA (2001) Effects of urbanization on streams of the Melbourne region, Victoria, Australia. I. Benthic macroinvertebrate communities. Freshw Biol 46: 535-551. doi:10.1046/j.1365-2427.2001.00690.x.

[9] Wang L, Lyons J, Kanehl P, Bannerman R (2001) Impacts of urbanization on stream habitat and fish across multiple spatial scales. Environ Manag 28: 255-266. doi:10.1007/s0026702409. PubMed: 11443388. 10.1007/s0026702409 11443388

[10] Sonneman JA, Walsh CJ, Breen PF, Sharpe AK (2001) Effects of urbanization on streams of the Melbourne region, Victoria, Australia. II. Benthic diatom communities. Freshw Biol 46: 553-565. doi:10.1046/j.1365-2427.2001.00689.x.

[11] Paul MJ, Meyer JL (2008) Streams in the urban landscape. Urban Ecology: 207-231.

[12] Schueler TR (1992) Mitigating the adverse impacts of urbanization on streams: a comprehensive strategy for local government. Watershed Restoration Sourcebook, Publication 92701: 21–31

[13] Kennen JG, Chang M, Tracy BH, Brown L, Gray R et al. (2005). Effects of landscape change on fish assemblage structure in a rapidly growing metropolitan area in North Carolina, USA. Bethesda MD: American Fisheries Society USA pp. 39-52.

[14] Walsh CJ, Waller KA, Gehling J, Mac Nally R (2007) Riverine invertebrate assemblages are degraded more by catchment urbanisation than by riparian deforestation. Freshw Biol 52: 574-587. doi:10.1111/j.1365-2427.2006.01706.x.

[15] Cooper SD, Diehl S, Kratz K, Sarnelle O (1998) Implications of scale for patterns and processes in stream ecology. Aust J Ecol 23: 27-40. doi:10.1111/j.1442-9993.1998.tb00703.x.

[16] Thorp JH, Thoms MC, Delong MD (2006) The riverine ecosystem synthesis: biocomplexity in river networks across space and time. River Res Applications 22: 123-147. doi:10.1002/rra.901.

[17] Allan JD (2004) Landscapes and riverscapes: the influence of land use on stream ecosystems. Annu Rev Ecol Evol Syst 35: 257-284. doi:10.1146/annurev.ecolsys.35.120202.110122.

[18] van der Ree R, McCarthy MA (2005) Inferring persistence of indigenous mammals in response to urbanisation. Anim Conserv 8: 309-319. doi:10.1017/S1367943005002258.

[19] Hamer AJ, McDonnell MJ (2008) Amphibian ecology and conservation in the urbanising world: a review. Biol Conserv 141: 2432-2449. doi:10.1016/j.biocon.2008.07.020.

[20] Hamer AJ, Parris KM (2011) Local and landscape determinants of amphibian communities in urban ponds. Ecol Applications 21: 378-390. doi:10.1890/10-0390.1. PubMed: 21563570. 10.1890/10-0390.1 21563570

[21] Parris KM (2006) Urban amphibian assemblages as metacommunities. J Anim Ecol 75: 757-764. doi:10.1111/j.1365-2656.2006.01096.x. PubMed: 16689958. 16689958 10.1111/j.1365-2656.2006.01096.x

[22] Pillsbury FC, Miller JR (2008) Habitat and landscape characteristics underlying anuran community structure along an urban-rural gradient. Ecol Applications 18: 1107-1118. doi:10.1890/07-1899.1. PubMed: 18686575. 10.1890/07-1899.1 18686575

[23] Hamer AH, Lane SL, Mahony MM (2002) The role of introduced mosquitofish (*Gambusia holbrooki*) in excluding the native green and golden bell frog (*Litoria aurea*) from original habitats in south-eastern Australia. Oecologia 132: 445-452. doi:10.1007/s00442-002-0968-7. 28547423 10.1007/s00442-002-0968-7

[24] Barrett K, Guyer C (2008) Differential responses of amphibians and reptiles in riparian and stream habitats to land use disturbances in western Georgia, USA. Biol Conserv 141: 2290-2300. doi:10.1016/j.biocon.2008.06.019.

[25] Barrett K, Helms BS, Guyer C, Schoonover JE (2010) Linking process to pattern: causes of stream-breeding amphibian decline in urbanized watersheds. Biol Conserv 143: 1998-2005. doi:10.1016/j.biocon.2010.05.001.

[26] Lane A, Burgin S (2008) Comparison of frog assemblages between urban and non-urban habitats in the upper Blue Mountains of Australia. Freshw Biol 53: 2484-2493. doi:10.1111/j.1365-2427.2008.02068.x.

[27] Riley SPD, Busteed GT, Kats LB, Vandergon TL, Lee LFS et al. (2005) Effects of urbanization on the distribution and abundance of amphibians and invasive species in southern California streams. Conserv Biol 19: 1894-1907. doi:10.1111/j.1523-1739.2005.00295.x.

[28] Vannote RL, Minshall GW, Cummins KW, Sedell JR, Cushing CE (1980) The river continuum concept. Can J Fish Aquat Sci 37: 130-137. doi:10.1139/f80-017.

[29] Ficetola GF, Marziali L, Rossaro B, De Bernardi F, Padoa-Schioppa E (2011) Landscape-stream interactions and habitat conservation for amphibians. Ecol Applications 21: 1272-1282. doi:10.1890/10-0431.1. PubMed: 21774429. 10.1890/10-0431.1 21774429

[30] Walsh CJ, Kunapo J (2009) The importance of upland flow paths in determining urban effects on stream ecosystems. J North American Benthological Society 28: 977-990. doi:10.1899/08-161.1.

[31] Wang L, Lyons J, Kanehl P (2003) Impacts of urban land cover on trout streams in Wisconsin and Minnesota. Trans Am Fish Soc 132: 825-839. doi:10.1577/T02-099.

[32] Lauck B (2005) The impact of recent logging and pond isolation on pond colonization by the frog *Crinia signifera* . Pac Conserv Biol 11: 50-56.

[33] Canessa S, Heard GW, Parris KM, McCarthy MA (2012) Integrating variability in detection probabilities when designing wildlife surveys: a case study of amphibians from south-eastern Australia. Biodivers Conserv 21: 729-744. doi:10.1007/s10531-011-0211-0.

[34] Heard GW, Robertson P, Scroggie MP (2006) Assessing detection probabilities for the endangered growling grass frog (*Litoria raniformis*) in southern Victoria. Wildl Res 33: 557-564. doi:10.1071/WR04080.

[35] Barker J, Grigg GC, Tyler MJ (1995) A field guide to Australian Frogs. Sydney: Surrey Beatty.

[36] Elith J, Phillips SJ, Hastie T, Dudík M, Chee YE et al. (2011) A statistical explanation of MaxEnt for ecologists. Divers Distrib 17: 43-57. doi:10.1111/j.1472-4642.2010.00725.x.

[37] Trombulak SC, Frissell CA (2001) Review of ecological effects of roads on terrestrial and aquatic communities. Conserv Biol 14: 18-30.

[38] Parris KM, McCarthy MA (1999) What influences the structure of frog assemblages at forest streams? Aust J Ecol 24: 495-502. doi:10.1046/j.1442-9993.1999.00989.x.

[39] Elith J, Leathwick JR, Hastie T (2008) A working guide to boosted regression trees. J Anim Ecol 77: 802-813. doi:10.1111/j.1365-2656.2008.01390.x. PubMed: 18397250. 18397250 10.1111/j.1365-2656.2008.01390.x

[40] R Development Core Team (2011) R: a language and environment for statistical computing. Vienna, Austria: R Foundation for Statistical Computing.

[41] Shipley B (2002) Cause and correlation in biology: a user’s guide to path analysis, structural equations and causal inference. Cambridge University Press.

[42] Plummer M (2003). JAGS: a program for analysis of Bayesian graphical models using Gibbs sampling. Austria: Vienna.

[43] Spiegelhalter DJ, Best NG, Carlin BP, van der Linde A (2002) Bayesian measures of model complexity and fit (with discussion). J R Stat Soc B Stat Methodol (Statistical Methodology) 64: 583-639

[44] Smith AM, Green DM (2005) Dispersal and the metapopulation paradigm in amphibian ecology and conservation: are all amphibian populations metapopulations? Ecography 28: 110-128. doi:10.1111/j.0906-7590.2005.04042.x.

[45] Lichstein JW, Simons TR, Shriner SA, Franzreb KE (2002) Spatial autocorrelation and autoregressive models in ecology. Ecol Monogr 72: 445-463. doi:10.1890/0012-9615(2002)072[0445:SAAAMI]2.0.CO;2.

[46] Heard GW (2010) Pattern, process and the conservation of threatened amphibian metapopulations. Bundoora. Victoria, Australia: La Trobe University, Ph.D. Thesis

[47] Hamer AJ, Smith PJ, McDonnell MJ (2012) The importance of habitat design and aquatic connectivity in amphibian use of urban stormwater retention ponds. Urban Ecosyst 15: 451-471. doi:10.1007/s11252-011-0212-5.

[48] Groffman PM, Bain DJ, Band LE, Belt KT, Brush GS et al. (2003) Down by the riverside: urban riparian ecology. Front Ecol Environ 1: 315-321. doi:10.1890/1540-9295(2003)001[0315:DBTRUR]2.0.CO;2.

[49] Fahrig L, Rytwinski T (2009) Effects of roads on animal abundance: an empirical review and synthesis. Ecol Soc 14: 1-20.

[50] Eigenbrod F, Hecnar SJ, Fahrig L (2008) The relative effects of road traffic and forest cover on anuran populations. Biol Conserv 141: 35-46. doi:10.1016/j.biocon.2007.08.025.

[51] Walsh CJ, Fletcher TD, Burns MJ (2012) Urban stormwater runoff: a new class of environmental flow problem. PLOS ONE 7: e45814. doi:10.1371/journal.pone.0045814. PubMed: 23029257. 23029257 10.1371/journal.pone.0045814 PMC3446928

[52] Anstis M (2002) Tadpoles of south-eastern Australia: a guide with keys. Sydney, New South Wales, Australia: Reed New Holland.

[53] Brabec E (2002) Impervious surfaces and water quality: a review of current literature and its implications for watershed planning. J Plan Lit 16: 499-514. doi:10.1177/088541202400903563.

[54] Ranvestel AW, Lips KR, Pringle CM, Whiles MR, Bixby RJ (2004) Neotropical tadpoles influence stream benthos: evidence for the ecological consequences of decline in amphibian populations. Freshw Biol 49: 274-285. doi:10.1111/j.1365-2427.2004.01184.x.

[55] Wassersug RJ (1975) The adaptive significance of the tadpole stage with comments on the maintenance of complex life cycles in Anurans. Am Zool 15: 405-417.

[56] Altig R, Whiles MR, Taylor CL (2007) What do tadpoles really eat? Assessing the trophic status of an understudied and imperiled group of consumers in freshwater habitats. Freshw Biol 52: 386-395. doi:10.1111/j.1365-2427.2006.01694.x.

[57] Taylor SL, Roberts SC, Walsh CJ, Hatt BE (2004) Catchment urbanisation and increased benthic algal biomass in streams: linking mechanisms to management. Freshw Biol 49: 835-851. doi:10.1111/j.1365-2427.2004.01225.x.

[58] Price SJ, Dorcas ME, Gallant AL, Klaver RW, Willson JD (2006) Three decades of urbanization: estimating the impact of land-cover change on stream salamander populations. Biol Conserv 133: 436-441. doi:10.1016/j.biocon.2006.07.005.

[59] Victorian. Government (2009) Delivering Melbourne's newest sustainable communities. In: Department of Planning and Community Development, editor. Melbourne, Australia

[60] McKenney BA, Kiesecker JM (2010) Policy development for biodiversity offsets: a review of offset frameworks. Environ Manag 45: 165-176. doi:10.1007/s00267-009-9396-3. PubMed: 19924472. 10.1007/s00267-009-9396-3 19924472

